# Clinical Epidemiology Characteristics and Etiology of Febrile Neutropenia in Children: Analysis of 421 Cases

**DOI:** 10.3390/hematolrep14030034

**Published:** 2022-08-01

**Authors:** Sang Ngoc Nguyen, Lam Tung Vu, Quang Van Vu, Tham Thi Tran, Vi Tuong Thi Dinh

**Affiliations:** Faculty of Medicine, Haiphong University of Medicine and Pharmacy, Haiphong 18000, Vietnam; tunglamvghy@gmail.com (L.T.V.); vvquang@hpmu.edu.vn (Q.V.V.); tttham@hpmu.edu.vn (T.T.T.); hoatuanhconno@gmail.com (V.T.T.D.)

**Keywords:** children, fever, neutropenia, febrile neutropenia

## Abstract

The congenital immune system includes neutrophils, which perform a variety of functions. Congenital and acquired neutropenia are rare illnesses with an underestimated prevalence in children. The aim of this study is to examine the epidemiology and etiology of febrile neutropenia in children at Haiphong Children’s Hospital, Haiphong, Vietnam. **Methods:** A cross-sectional study was carried out on 421 febrile neutropenia children. Clinical and laboratory characteristics were examined. **Results:** The median age (IQR) was 25.0 (12.5–59.5) months. The male-to-female ratio was 1.35/1. There were twice as many children living in the suburbs (66.98%) as in urban areas (33.02%). The mean (SD) temperature at admission was 38.50 ± 0.59 °C. Diagnosed causes associated with neutropenia included acute respiratory infections 250 (59.45%), gastrointestinal infections 68 (16.1%), erythema 37 (8.79%), acute leukemia 15 (3.56%), urinary tract infection 5 (1.19%), and encephalitis/meningitis 4 (0.95%). Viral etiology accounted for 61.52% (259): *influenza type A*—50.19% (130), *influenza type B*—31.27% (81), *dengue virus*—14.67% (38), *measles virus* 1—93% (5), rotavirus—1.54% (4), and *EBV*—0.4% (1). Twenty-five patients (5.94%) were found to have bacteria in their cultures, with *Streptococcus pneumonia* being the most common (eight patients; 32%). **Conclusions:** Febrile neutropenia was common in children under 2 years old. Primary clinical manifestations were acute upper respiratory tract infections, and viruses most commonly caused febrile neutropenia. Further studies with larger sample sizes are needed to determine the cause of febrile neutropenia.

## 1. Introduction

Neutrophils are an important component of the congenital immune system that have a range of vital responsibilities [1,2]. The severity of an infection is typically proportional to the number of neutrophils present. There is an increased risk of infection when the neutrophil count is less than 0.5 × 10^9^ cells/L for an extended period of time [3,4,5]. In contrast, when the neutrophil count exceeds 0.5 × 10^9^ cells/L or the duration of neutropenia decreases, the risk is significantly reduced [6]. Both congenital and acquired neutropenia are rare diseases whose prevalence in children is underestimated [7]. However, many children with moderate-to-severe neutropenia have a benign condition, which suggests that there are other factors to consider—such as the rate of onset, time taken to reduce reserve bone marrow neutrophils, monocyte count, and phagocytic cell activity—that may affect infection [8,9,10]. The clinical presentation of neutropenia varies depending on underlying medical conditions, with fever being a typical symptom in these patients [11]. Neutropenia can be caused by a number of factors. Genetic, pharmacological, and post-infectious factors are all major culprits [12,13,14]. According to Dale and Bolyard’s 2017 study on common respiratory pathogens among febrile neutropenic children, only two-thirds of the causes of fever were detected in all of the studied children, with viruses accounting for 51.8%, bacteria for 11.4%, and fungi for 3% [15]. The most common viral etiology was rhinovirus, followed by respiratory syncytial virus and coronavirus [16].

The aim of this study was to analyze the epidemiology and etiology of febrile neutropenia in children treated at Haiphong Children’s Hospital, Haiphong, Vietnam.

## 2. Materials and Methods

A descriptive cross-sectional study was carried out for one year: from 1 January 2019 to 31 December 2019. The study site was Haiphong Children’s Hospital in Haiphong City, Vietnam.

### 2.1. Inclusion Criteria

Participants in the study were children who suffered from febrile neutropenia and were treated in Haiphong Children’s Hospital. All of them were diagnosed based on the recommendations of the Italian Association of Pediatric Hematology and Oncology (Associazione Italiana Emato-Oncologia Pediatrica, AIEOP) [7].

### 2.2. Exclusion Criteria

These cases were excluded:

- Patients undergoing chemotherapy, which causes neutropenia.
- Patients suffering from systemic diseases such as systemic lupus erythematosus (SLE).

### 2.3. Sample Size

All patients who fit the criteria for the study were included.

### 2.4. Methodology

This format was used to collect all of the data:

1. Individual’s demographic: age, sex;
2. Family history: occurrence of other neutropenia cases;
3. Viral and bacterial infections were the focus of the personal history (onset, number, kind, site, frequency and severity, and cyclic pattern of infections), as well as assumptions about medication (type, duration, and dose);
4. Clinical examination: At the entrance and during follow-up, all organs were examined, including the respiratory, gastrointestinal, urinary, and dermatological systems, as well as the signs and symptoms of sepsis;
5. Laboratory examination: complete blood cells, C-reactive protein, and/or DNA/RNA analysis of some viral pathogens, including *type A influenza, type B influenza, dengue virus, measles virus, rotavirus,* and *Epstein–Barr virus;*
6. Investigating the cause of neutropenia: finding comorbidities, such as respiratory infections, gastrointestinal infections, urinary tract infections, typhus, and acute leukemia.

### 2.5. Laboratory Methodology

#### Bacteria Identification

##### Collecting Samples

Throat swab culture: Throat cultures were collected by rubbing a sterile swab tip on the surface of one or both tonsils, the tonsillar pillars, or the posterior pharyngeal wall.

Urine culture: A single organism cultured from a suprapubic aspirate (SPA), transurethral catheterization, or clean-catch midstream specimen (CCM).

Stool culture: Stool specimens were collected in aseptic storage bins and carefully handled following standard laboratory procedures.

Cerebrospinal fluid (CSF) culture: Lumbar punctures were carried out with a sterile technique, which included skin preparation with povidone iodine, sterile drapes, and surgical masks.

Inoculation of pus on the skin: An aspirate from a previously undrained abscess was made with a sterile syringe, and any air bubbles were expelled. The sample volume was 1–5 mL.

Blood culture: Blood samples were collected prior to the administration of antibiotics and at the onset of clinical symptoms.

All kinds of samples at our institution are cultured on blood and chocolate agar. Incubation conditions: 35–37 °C, atmosphere 5–7% CO_2_ and duration: 15–18 h. Any positive cultures were subsequently Gram stained.

##### Virus Identification

We used a real-time PCR procedure based on automated specimen extraction and multiplex amplification. Primers and hydrolysis probes were obtained from the literature or developed in our laboratory. The following viral agents were analyzed: *influenza viruses A and B*, *dengue virus* (DENV), *measles virus*, *rotavirus*, and *Epstein–Barr virus* (EBV).

### 2.6. Data Analysis

The categorical data are presented as the number of cases and percentages. The continuous data are presented as mean (standard deviation [SD]) or median (interquartile range [IQR]) as appropriate.

Pearson’s chi-square test or Fisher’s exact test were used for analyzing the association between categorical variables. All analyses were conducted using Statistical Package for Social Sciences (SPSS) version 26.0, and a *p*-value of less than 0.05 was considered significant.

## 3. Results

**Figure 1:**
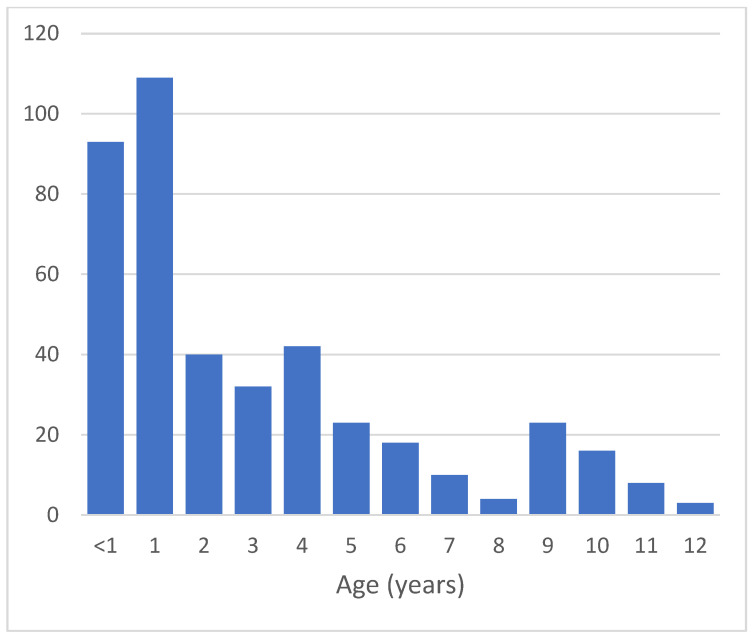
Age distribution of children with febrile neutropenia.

Over the one-year period from 1 January 2019 to 31 December 2019, 47,761 children were admitted to Haiphong Children’s Hospital, Haiphong, Vietnam, and 421 of these were hospitalized due to febrile neutropenia, accounting for 0.88%. Table 1 illustrates the demographic features of all studied patients. A total of 242 children (57.5%) were male, and 179 (42.5%) were female. The age of children with febrile neutropenia was notably young. In detail, children aged from 12 months to 24 months contributed the largest share, at 25.9%, followed by those aged under 1 year (22.1%) (see Figure 1). The median age (IQR) was 25.0 (12.5–59.5) months.

Most patients had a fever (Table 2). Three groups showed a rise in temperatures: 155 (36.8%) children in the first group had a temperature ranging from 38 °C to under 38.5 °C; 139 (33%) children in the second group had a temperature ranging from 38.5 °C to under 39 °C, and 127 (30.2%) children in the third group had a temperature of 39 °C or above. The mean and SD was 38.5 ± 0.6 °C, and the median and IQR duration of fever was 4.0 (3.0–5.0) days. Upon clinical presentation, of the 250 (59.38%) children with febrile neutropenia, 156 had upper respiratory tract infections, and the remaining 64.4% had lower respiratory tract infections. Our results show that 68 (16.15%) patients had gastrointestinal symptoms, including vomiting and watery stools. Urinary tract infection, sepsis, and encephalitis meningococcal infection were less common, accounting for 1.19%, 1.19%, and 0.95%, respectively. There were 15 febrile neutropenic patients who had acute leukemia, including 11 children with acute lymphocytic leukemia and 4 children with acute myeloid leukemia (see Table 2). In terms of laboratory investigation, a complete blood count test was carried out in all 421 studied children (see Table 3). Only 48 of our patients (11.4%) had a neutrophil count of less than 0.5 × 10^9^/L. Those with neutrophil counts between 1 and 1.5 × 10^9^/L and those with counts between 0.5 and 1 × 10^9^/L had almost similar proportions. The mean (SD) neutrophil count was 0.95 ± 0.33 × 10^9^/L. The majority of children with febrile neutropenia were anemic (85.04%). Only 17 (4.04%) of the 421 individuals had thrombocytopenia 79.10% of 421 serum C-reactive protein results were in the normal range. Only 79 (22.01%) patients had X-ray images of pneumonia,

Table 4 shows the results of bacterial cultures. Fourteen (51.8%) of the twenty-seven throat swab samples contained microorganisms (51.85%). Meanwhile, only five urine samples were collected from patients, all of which were positive. A stool culture was positive in four patients (100%), a skin pus culture was positive in one patient (100%), and blood culture was positive in one patient (20%). Table 5 presents all bacteria, and viral agents detected in children with febrile neutropenia. *Type A influenza* infected 130 of them (50.19%), followed by *Type B influenza*, which infected 81 (31.27%). The number of patients with *dengue virus* (DENV) was lower, with 38 (14.67%) cases. *Measles virus*, *rotavirus*, and *Epstein–Barr virus* (EBV) were less common, with 1.93%, 1.54%, and 0.4%, respectively. Our findings revealed that Gram-positive bacteria were more prevalent in 25 culture tests, with eight (32%) cases containing *Streptococcus pneumonia* and five (20%) cases containing *Staphylococcus aureus*.

## 4. Discussion

According to our demographic data, febrile neutropenia was observed in both groups children of every age, and we noted that the age of children diagnosed with febrile neutropenia was very young, with 48% under 24 months old. Angelino et al. indicated that children aged under 12 months accounted for the highest proportion, at 48%, followed by children aged from 12 to 24 months (24%) [17]. We did not find any research that explained why febrile neutropenia commonly occurred in children aged under 2 years. In addition, we noticed that the number of males was higher than that of females, and the male-to-female ratio was 1.35/1, which followed the same trend as previous studies. In his investigation, Aldemir-Kocabas [16] found that the male–female ratio was 1.6/1, while Angelino [17] found that it was 1.21.

Regarding the clinical characteristics, we noted that the mean temperature was 38.5 ± 0.6 °C, and the median duration of fever was 4.0 (3.00–5.00) days. Of the patients, 30.2% had a temperature greater than 39 °C, similar to Das’s study [13], with 32% of children having a temperature above 39 °C. Febrile neutropenia was also known as a common complication of leukemia, especially when patients received chemotherapy [18,19]. We found that 15 (3.5%) children diagnosed with leukemia had febrile neutropenia. Phillips [14] reported that 52% of children with leukemia had febrile neutropenia, much higher than in our study.

Our findings show that the mean neutrophil count was 0.95 ± 0.33 × 10^9^/L, with 47.3% of children having mild neutropenia and 41.3% of children having moderate neutropenia. Tantawy’s research [20,21] indicates a similar pattern, showing that the number of children with mild neutropenia was the highest (45%). Tantawy also noted that the neutrophil count fluctuated between 0.1 and 1.28 × 10^9^/L, and the mean neutrophil count was 0.827 ± 0.4375 × 10^9^/L. Our data show that most patients had serum C-reactive protein results in the normal range (<12 mg/L). Patients with serum CRP of less than 12 mg/L were mainly assumed to have a viral infection, which is plausible since 61.52% of those with febrile neutropenia were positive with one of our viral tests. In the study by Avabratha [22], febrile neutropenic patients were divided into three groups, and their CRP was monitored from day 1 to day 7 of antibiotic treatment, and three groups of adults had a change in CRP from positive to negative. CRP is a valuable marker for assessing infection in neutropenic children and evaluating the effectiveness of antibiotic therapy.

Among children with febrile neutropenia, the majority of cases had a positive viral test, with 259 (61.52%) cases. In our study, respiratory viruses caused the most cases, including 130 (50.19%) cases of *type A influenza*, followed by 81 (31.27%) cases of *type B Influenza*. We found that respiratory viruses accounted for the most instances, including 130 (50.19%) cases of *type A influenza*, followed by *type B influenza*, with 81 (31.27%) cases. Conversely, measles, rotavirus, and Epstein–Barr virus were less frequent. Aldemir-Kocabas [16] and Suryadevara demonstrated a similar pattern, with respiratory virus percentages of 51.8% and 52%, respectively. According to Walkovich and Boxer [23], the most common cause of acute neutropenia among children is infectious diseases, and viruses such as *RSV*, *Varicella*, *Influenza type A* and *type B*, *measles*, and *EBV* are the primary agents that commonly cause neutropenia. We also found that bacteria were the primary cause of neutropenia in 25 cases. *Streptococcus pneumonia* (eight cases) and *Staphylococcus aureus* (five cases) were among the most common Gram-positive bacteria that were isolated. The study also discovered Gram-negative bacteria such as *Moraxella catarrhalis*, *Hemophilus influenza*, *Escherichia coli*, and *Pseudomonas aeruginosa*. Segel’s research [24] indicated that 45% of causes were Gram-positive, but these findings were much greater, including *Staphylococcus aureus*, and *Streptococcus pneumonia*. Moreover, Avabratha’s research [22] found that 15 of 33 Indian patients had bacterial infections, with Gram-positive bacteria accounting for seven cases (46.7%) and Gram-negative bacteria accounting for eight cases (53.8%). In this study, *Staphylococcus aureus* and *Escherichia coli* were the most common bacteria, each with four instances, followed by *Pseudomonas* *aeruginosa*, which had three instances.

## 5. Conclusions

Febrile neutropenia was common in children under the age of 2 years. The primary clinical manifestation was an acute upper respiratory tract infection. The most common cause of neutropenia was a virus. Other bacteria and acute leukemia were also present. To develop a proper diagnosis and treatment plan for patients suffering from febrile neutropenia, physicians must understand the epidemiology and etiology of the disease. Various treatment regimens may be necessary as the range of pathogenic bacteria expands. Our findings shed light on the cause of febrile neutropenia in order to aid clinicians in the diagnosis, treatment, and prognosis of diseases. Finally, our findings highlight the importance of further research in different groups of children with neutropenia in order to assess the predictive potential of risk factors for severe invasive bacterial infection. Patients can benefit from a selective approach if a practical and reliable categorization system is developed, which may improve their quality of life.

## Figures and Tables

**Table 1:** The demographics of hospitalized children with febrile neutropenia (*n* = 421).

Variables	*n* (%)
Sex	
Male	242 (57.5)
Female	179 (42.5)
Geographical features	
Rural	282 (66.98)
Urban	139 (33.02)

**Table 2:** Clinical characteristics of studied patients (*n* = 421).

Variables	*n* (%)
Temperature at admission	
38–<38.5 °C	155 (36.8)
38.5–<39 °C	139 (33)
≥39 °C	127 (30.2)
Respiratory symptoms	250 (59.38)
Upper respiratory tract infection	156 (62.4)
Lower respiratory tract infection	94 (37.6)
Gastrointestinal symptoms	68 (16.15)
Vomiting	33 (48.52)
Watery stools	34 (50)
Bloody stools	1 (1.47)
Rash	37 (8.79)
Acute leukemia	15 (3.5)
Acute lymphocytic leukemia	11 (73.34)
Acute myeloid leukemia	4 (26.66)
Urinary tract infection	5 (1.19)
Sepsis	5 (1.19)
Encephalitis meningococcal infection	4 (0.95)

**Table 3:** Investigation results of children with febrile neutropenia (*n* = 421).

Variables	*n* (%)
Neutrophil count	
<0.5 × 10^9^/L	48 (11.4)
0.5–1 × 10^9^/L	174 (41.3)
1–1.5 × 10^9^/L	199 (47.3)
Hemoglobin	
<80 g/L	12 (2.85)
80–100 g/L	51 (12.11)
>100 g/L	358 (85.04)
Platelet count	
<100 × 10^9^ /L	17 (4.04)
100–400 × 10^9^ /L	395 (93.83)
>400 × 10^9^ /L	9 (2.13)
Serum C-reactive protein	
≥12 mg/L	88 (20.90)
<12 mg/L	333 (79.10)
Chest X-ray	
Normal	280 (77.99)
Pneumonia	79 (22.01)

Reference range: Neutrophil count: 2.5−7.5 × 10^9^/L, Hemoglobin: 120−160 g/L, Platelet count: 15−450 × 10^9^/L, Serum C-reactive protein: 0−10 mg/L.

**Table 4:** Culture results to find the causes of febrile neutropenia.

Culture Results	Number of Patients (%)
Throat Swab culture—positive	14/27 (51.85)
Urine culture—positive	5/5 (100)
Stool culture—positive	4/4 (100)
Cerebrospinal fluid (CSF) culture—positive	0/4 (0)
Inoculation of pus on the skin—positive	1/1 (100)
Blood culture—positive	1/5 (20)

**Table 5:** Bacterial and viral agents found in children with febrile neutropenia.

Bacterial and Viral Agents	*n* (%)
***Bacteria***	25
Gram-positive bacteria	
*Streptococcus pneumonia*	8 (32.00)
*Staphylococcus aureus*	5 (20.00)
Gram-negative bacteria	
*Moraxella catarrhalis*	4 (16.00)
*Hemophilus influenza*	3 (12.00)
*Escherichia coli*	4 (16.00)
*Pseudomonas aeruginosa*	1 (4.00)
***Virus***	259 (61.52)
*Type A influenza*	130 (50.19)
*Type B influenza*	81 (31.27)
*Dengue virus (DENV)*	38 (14.67)
*Measles virus*	5 (1.93)
*Rotavirus*	4 (1.54)
*Epstein–Barr virus (EBV)*	1 (0.4)

## Data Availability

Data available on request due to restrictions, such as privacy or ethical issues. The data presented in this study are available on request from the corresponding author. The data are not publicly available due to the lack of an accessible server.

## Abbreviations

CRP	C-reactive protein
EBV	Epstein–Barr virus
RSV	respiratory syncytial virus
